# Supplementary material for: The Luria-Nebraska Neuropsychological Battery Neuromotor Tasks: From Conventional to Image-Derived Measures
Source: Brain Sci. 2022 Jun 8;12(6):757. doi: 10.3390/brainsci12060757 (PMC9221253; doi:10.3390/brainsci12060757)
Supplement: Supplementary file 1 [file brainsci-12-00757-s001.zip › brainsci-1714990-supplementary.pdf]

SUPPLEMENTAL MATERIAL

Title: The Luria-Nebraska Neuropsychological Battery neuromotor tasks: from conventional to image-derived measures

Daniele Corbo, Donatella Placidi, Roberto Gasparotti, Robert Wright, Donald R Smith, Roberto G Lucchini,Megan Horton, Elena Colicino

Table S1. Pearson correlation coefficient between the conventional and the image-derived Luria-Nebraska Neuropsychological Battery (LNNB) task scores among 46 PHIME participants. Task 1: the dominant hand clench, Task2: the finger-thumb touching with the dominant hand, Task 3: the non-dominant hand clench, Task 4: the finger-thumb touching with non-dominant hand, Task 5 alternative hand clench.

LNNB tasks	rho	95% Confidence Interval
Task 1	0.74	(0.57; 0.85)
Task 2	0.72	(0.55; 0.84)
Task 3	0.70	(0.51; 0.82)
Task 4	0.71	(0.53; 0.83)
Task 5	0.72	(0.54; 0.83)

Table S2. Area under the Receiver Operating Characteristic (ROC) curve (AUC) and 95% Confidence Interval (95%CI) of each Luria-Nebraska Neuropsychological Battery (LNNB) task, classifying participants with lower mobility (LNNB score < population mean levels). Task 1: the dominant hand clench, Task2: the finger-thumb touching with the dominant hand, Task 3: the non-dominant hand clench, Task 4: the finger-thumb touching with non-dominant hand, Task 5 alternative hand clench.

LNNB tasks	AUC	95%CI	Specificity (70% sensitivity)
Task 1	0.73	(0.6; 0.86)	0.71
Task 2	0.83	(0.72; 0.94)	0.83
Task 3	0.75	(0.62; 0.88)	0.71
Task 4	0.70	(0.57; 0.83)	0.63
Task 5	0.72	(0.58; 0.85)	0.67

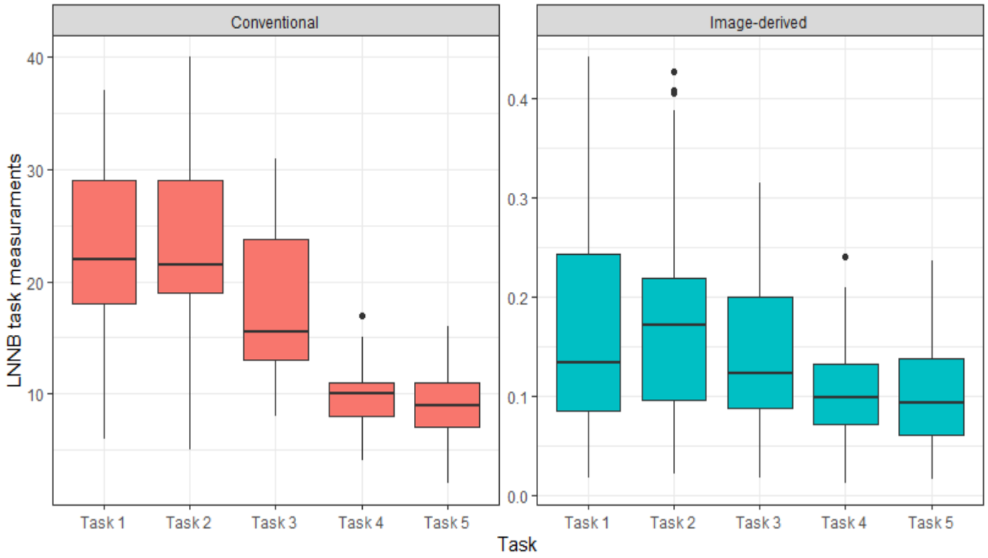

Figure S1. Boxplots of the Luria-Nebraska Neuropsychological Battery (LNNB) task scores: a) conventional LNNB measurements; b) the image-derived LNNB measurements.

Task 1: the dominant hand clench, Task2: the finger-thumb touching with the dominant hand, Task 3: the non-dominant hand clench, Task 4: the finger-thumb touching with non-dominant hand, Task 5 alternative hand clench.
